# Precisely Designed Morphology and Surface Chemical Structure of Fe-N-C Electrocatalysts for Enhanced Oxygen Reaction Reduction Activity

**DOI:** 10.3390/molecules29163785

**Published:** 2024-08-10

**Authors:** Zirun Chen, Yuang Xiong, Yanling Liu, Zhanghongyuan Wang, Binbin Zhang, Xingtang Liang, Xia Chen, Yanzhen Yin

**Affiliations:** 1 Guangxi Key Laboratory of Green Chemical Materials and Safety Technology, Beibu Gulf University, Qinzhou 535011, Chinazhangbb@bbgu.edu.cn (B.Z.);; 2 Guangxi Key Laboratory of Beibu Gulf Marine Biodiversity Conservation, College of Marine Sciences, Beibu Gulf Ocean Development Research Center, Beibu Gulf University, Qinzhou 535011, China

**Keywords:** tunable morphology, tailored surface chemical structure, Fe-N-C electrocatalysts, ORR

## Abstract

Fe-N-C materials have been regarded as one of the potential candidates to replace traditional noble-metal-based electrocatalysts for the oxygen reduction reaction (ORR). It is believed that the structure of carbon support in Fe-N-C materials plays an essential role in highly efficient ORR. However, precisely designing the morphology and surface chemical structure of carbon support remains a challenge. Herein, we present a novel synthetic strategy for the preparation of porous carbon spheres (PCSs) with high specific surface area, well-defined pore structure, tunable morphology and controllable heteroatom doping. The synthesis involves Schiff-based polymerization utilizing octaaminophenyl polyhedral oligomeric silsesquioxane (POSS-NH_2_) and heteroatom-containing aldehydes, followed by pyrolysis and HF etching. The well-defined pore structure of PCS can provide the confinement field for ferroin and transform into Fe-N-C sites after carbonization. The tunable morphology of PCS can be easily achieved by changing the solvents. The surface chemical structure of PCS can be tailored by utilizing different heteroatom-containing aldehydes. After optimizing the structure of PCS, Fe-N-C loading on N,S-codoped porous carbon sphere (NSPCS-Fe) displays outstanding ORR activity in alkaline solution. This work paves a new path for fabrication of Fe-N-C materials with the desired morphology and well-designed surface chemical structure, demonstrating significant potential for energy-related applications.

## 1. Introduction

The ever-growing demand for clean and sustainable energy has directed the global scientific community towards the development of efficient electrocatalytic systems that can facilitate key energy conversion reactions. Among them, ORRs are pivotal in technologies ranging from fuel cells to metal-air batteries [1,2,3,4,5]. However, the widespread adoption of these technologies is often hindered by the limitations of existing electrocatalysts, which frequently suffer from issues such as high cost, limited durability, and suboptimal activity. Traditionally, noble metals, such as platinum and iridium, have been the benchmarks for electrocatalytic performance. However, poor durability and high cost have motivated researchers to seek alternative materials that can deliver comparable or superior performance with enhanced stability and lower cost [6,7]. In this regard, transition metal-based catalysts, particularly those derived from earth-abundant elements, have emerged as promising candidates [8,9,10,11]. However, the challenge lies in optimizing their electronic structure and surface morphology to achieve the desired catalytic properties.

One of the most significant advancements in this field has been the development of electrocatalysts with a well-designed nanostructure, which offer enhanced surface areas and tunable active sites [12,13,14]. Moreover, the advent of Fe-N-C electrocatalysts has further pushed the boundaries of catalytic efficiency by maximizing the atom utilization and offering unique geometric and electronic effects [15]. Despite the consensus that Fe-N-C sites are the dominant active sites, it is worth noting that the morphology and surface chemical structure (B, S or P doping) of carbon support have great impact on the ORR reactivity of Fe-N-C sites [16,17,18]. Recently, massive studies have focused on fabrication of Fe-N-C electrocatalysts through pyrolysis of metal salt with N-contain carbon precursors, such as metal-organic frameworks [19,20,21], polymer [22,23,24] and carbon materials [25,26,27,28]. However, these methods are hardly tailored to the morphology of carbon support, which will hinder the exposure of Fe-N-C sites and lower the utilization efficiency of Fe-N-C sites. Moreover, the traditional tunable doping method by adding additional heteroatom-related sources is a high cost, complex process and it is hard to implant into the carbon framework homogeneously [29,30,31]. Therefore, it is urgently needed to develop a carbon support with large surface areas, tailored morphology and tunable heteroatom to produce efficient Fe-N-C electrocatalysts.

Herein, we develop a novel synthetic route for preparation of carbon support with high surface area, tailored morphology and tunable heteroatom doping. Key to this novel synthetic strategy are the well-designed monomer and solvents for Schiff-based polymerization. In this work, the homogeneous organic/inorganic molecular interface generated by polyhedral oligomericsilsesquioxane (POSS) can produce a broad distribution of micropores and small mesopores in carbon support after pyrolysis and acid etching. The morphology of carbon support can be controlled by choosing different polymerization solvents. Moreover, the heteroatom of carbon support can be well tailored by different heteroatom-containing aldehyde monomers. Remarkably, through a well-designed morphology and surface chemical structure of the carbon support, Fe-N-C loading on an N,S-codoped porous carbon sphere performs remarkable ORR activity.

## 2. Results and Discussion

**Scheme 1:**
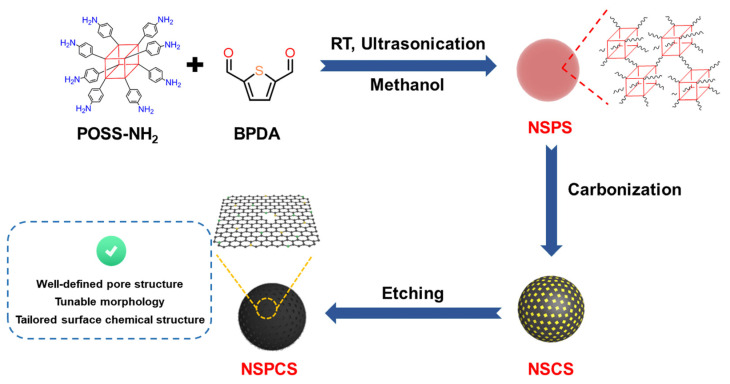
Schematic illustration of the synthesis of NSPCS materials.

The typical strategy for the synthesis of NSPCS is illustrated in Scheme 1. In brief, the POSS-NH_2_ and heteroatom-containing monomer, with two aldehyde groups in the molar ratio of 1:4, were mixed in methanol with acetic acid as catalyst, and with ultrasonication for 10 min, to form NSPS. After calcination in an N_2_ atmosphere, NSCS with homogeneously dispersed nano-silica domains was obtained. To remove the nano-silica template, NSCS is subsequently washed with HF, yielding the final product NSPCS. It is worth noting that an uneven interface between silica template and carbon source will cause a broad pore size distribution and a large pore size of the carbon support, leading to a poor loading of Fe-N-C sites and Fe aggregation. Our strategy involves creating a uniform carbon source/template interface by polycondensation of Octaaminophenyl polyhedral oligomeric silsesquioxane (POSS-NH_2_) and 2,5-thiophenedicarboxaldehyde. To verify the occurrence of polycondensation, the chemical structure of NSPS is studied by Fourier-transform infrared and Raman spectroscopy. As shown in Figure S1a, The FT–IR absorption peak at 1623 cm^−1^ is assignable to the amine (C=N) groups, which confirms the successful preparation of Schiff-based polymer NSPS [32,33]. Additionally, the carbonyl (C=O) group at 1699 cm^−1^ and nitrogen–hydrogen (N–H) bond at 3370 cm^−1^ suggest the presence of unreacted aldehyde and amine groups within the NSPS structure. Raman spectroscopy provides additional evidence for the polycondensation between POSS-NH_2_ and 2,5-thiophenedicarboxaldehyde, as illustrated in Figure S1b. Figure S2 illustrates that the NSPS exhibits a spherical morphology with an average diameter of approximately 450 nm. The X-ray diffraction (XRD) analysis of NSPS reveals a single broad peak at approximately 22.3°, revealing an amorphous structure with no discernible crystallographic order (Figure S3).

**Figure 1:**
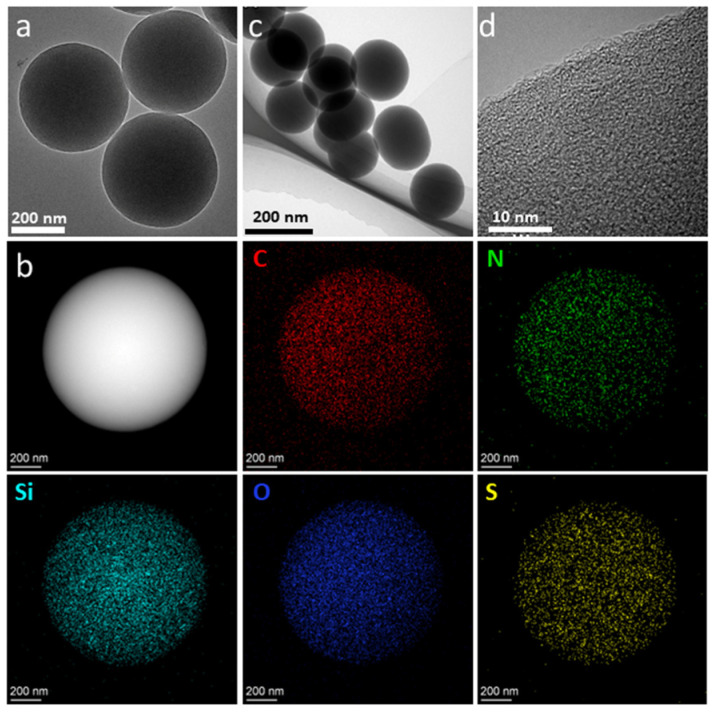
(**a**) TEM image of NSCS. (**b**) Elemental mapping image of NSCS revealing the good distribution of Si, O, S, N and C elements. (**c**,**d**) HRTEM images of NSPCS.

**Figure 2:**
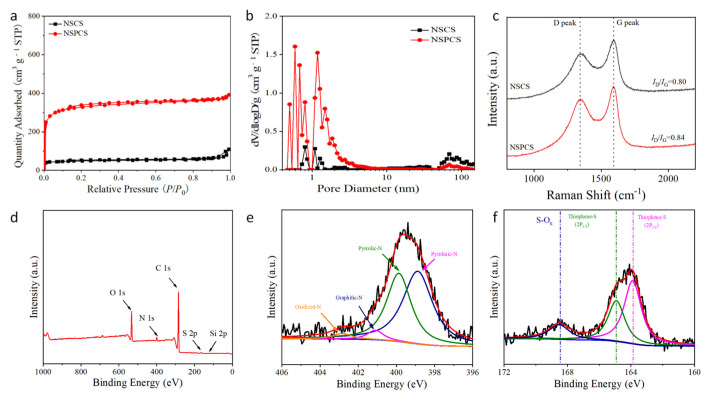
(**a**) N_2_ adsorption-desorption isotherms and (**b**) pore size distribution of NSCS and NSPCS. (**c**) Raman spectra of NSCS and NSPCS. (**d**) XPS survey spectrum of NSPCS. High-resolution XPS spectra of (**e**) N 1s and (**f**) S 2p for NSPCS.

To achieve NSPCS, NSPS was first pyrolyzed to produce NSCS. The NSPS demonstrates a high carbon yield and structure stability, attributed to the robust aromatic-silica hybrid framework and the rigid imine bond (Figure S4). Figure 1a,b reveal that no visible silica agglomeration was detected in the carbon matrix, and the energy dispersive spectrum (EDS) elemental mapping confirms the uniform distribution of Si, O, S and N atoms throughout the carbon framework. The findings indicate that a homogeneous carbon-silica molecular interface serves as an effective barrier to prevent silica aggregation and geometry destruction during carbonization. In addition, nitrogen adsorption–desorption analysis indicates that NSCS possesses a relatively high Brunauer–Emmett–Teller (BET) specific surface area of 166 m^2^ g^−1^ and narrow pore size distribution of 0.8~1.1 nm (Figure 2a). This feature provides access for HF to infiltrate the carbon matrix and fully erase the well-dispersed nano-silica from the NSCS. As shown in Figure 1c, the as-prepared NSPCS retains an inherited spherical architecture after HF etching. Furthermore, NSPCS possesses a disturbed structure of amorphous carbon (Figure 1d).

The removal efficiency of silica was investigated by thermogravimetric analysis (TGA) under an O_2_ stream. As shown in Figure S5, the silica content is significantly reduced from 42.8 wt% (NSCS) to 1.2 wt% (NSPCS), indicating that numerous well-defined pores were formed from the nano-silica template after HF etching. The pore characteristics of NSPCS, as shown in Figure 2a, display rapid nitrogen adsorption at low relative pressures and minimal hysteresis loops at high relative pressures, suggesting that micropores predominantly contribute to the pore structure within the carbon framework. The specific surface area and total pore volume of NSPCS reach 1114 m^2^ g^−1^ and 0.61 cm^3^ g^−1^, respectively. The pore size distribution curve reveals a broad micropore size distribution of 0.5~2.0 nm and a large number of small mesopores (<4.0 nm) formed after nano-silica removal (Figure 2b). The expanded micropores and small mesopores (1.3–4.0 nm) in NSPCS can provide a homogenous confinement region for ferroin (~1.3 nm), thus generating a well-dispersed Fe-N-C active site without Fe-based aggregation after high-temperature pyrolysis [34,35]. Additionally, the high ratio of small micropores (<1.3 nm) in NSPCS is beneficial to electrochemical activity [36,37].

To study the chemical structure of NSPCS, XRD patterns and Raman spectrum were utilized. As shown in Figure S6, the XRD pattern of NSPCS displays two broad peaks centered at approximately 24.9° and 43.3°, corresponding to the (002) and (100) reflections of carbon, respectively. These peaks suggest that NSPCS possesses a turbostratic structure characteristic of amorphous carbon. The Raman spectrum exhibits two distinct peaks at 1352 cm^−1^ and 1601 cm^−1^, associated with the D and G peaks of carbon, respectively (Figure 2c). Compared to NSCS, the higher *I*_D_/*I*_G_ ratio of NSPCS indicates the introduction of substantial defects after the removal of the nano-silica template. The surface element composition of NSPCS was characterized by X-ray photoelectron spectroscopy (XPS). The XPS survey spectrum indicates the existence of C, N, O, Si and S elements in NSPCS (as seen in Figure 2d and Table S1). The high-resolution N 1s spectrum was deconvoluted into four nitrogen species at binding energies of 398.9 eV, 399.9 eV, 401.1 eV, and 402.8 eV, corresponding to pyridinic-N, pyrrolic-N, graphitic-N, and oxidized-N, respectively (Figure 2e). The high-resolution S 2p spectrum in Figure 2f displays that the S species can be assigned to thiophene-S (163.9 eV and 164.9 eV) and S-O_x_ (168.5 eV).

It is worth noting that the morphology and surface chemical structure of an electrocatalyst play critical roles in ORR. Therefore, the controllable morphology and surface chemical structure of NSPCS were achieved via our strategy. As shown in Figure S8, NSPCB with bulk architecture was formed when using THF as the polycondensation solvent. Moreover, coral-like and worm-like morphology were formed by using 1,2-dichloroethane and acetone as reaction solvents, respectively. To modify the surface chemical structure of NSPCS, different types of aldehyde monomers were used to react with POSS-NH_2_. The XPS survey spectra of NPCS, NBPCS and NBrPCS reveal the existence of C, N, O, Si in NPCS, C, N, O, Si and B element in NBPCS, and C, N, O, Si and Br element in NBrPCS, respectively. The Ramen spectra display a higher *I*_D_/*I*_G_ ratio for NSPCS (0.84) than for NSPCB (0.74), suggesting that a spherical architecture can induce more defects in the carbon matrix (Figure S7b). The specific surface areas of NSPCB, NPCS, NBPCS, and NBrPCS are measured at 784 m^2^ g^−1^, 1173 m^2^ g^−1^, 1531 m^2^ g^−1^, and 1362 m^2^ g^−1^, respectively, with similar pore size distributions observed across these samples (Figure S9 and Table S2). These results indicate that our strategy can provide a tunable morphology and tailored surface chemical structure for carbon support, enabling the synthesis of highly efficient Fe-N-C electrocatalysts.

**Figure 3:**
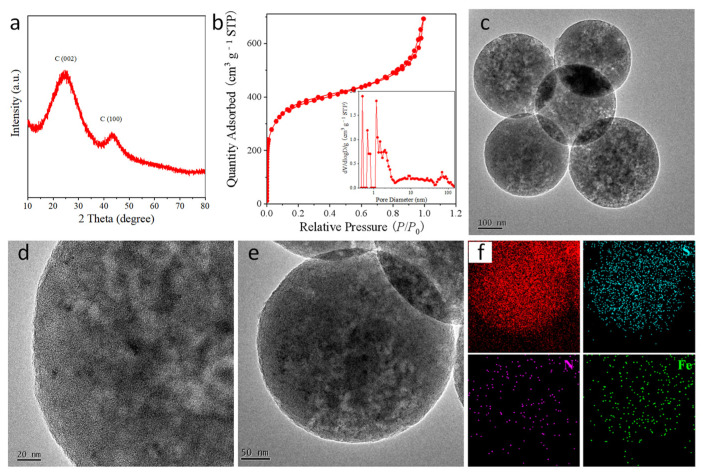
(**a**) XRD pattern of NSPCS-Fe. (**b**) N_2_ adsorption-desorption isotherms and pore size distribution of NSPCS-Fe. (**c**–**e**) HRTEM of NSPCS-Fe. (**f**) Elemental mapping image of NSPCS-Fe revealing the good distribution of C, S, N and Fe elements.

**Figure 4:**
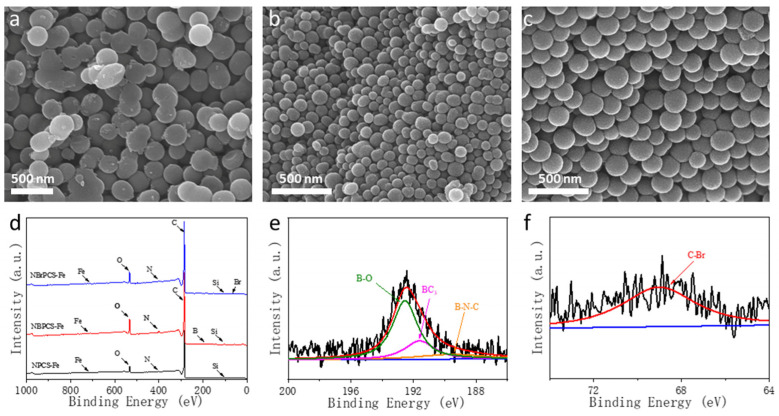
SEM images of (**a**) NPCS-Fe, (**b**) NBPCS-Fe and (**c**) NBrPCS-Fe. (**d**) XPS survey spectra of NPCS-Fe, NBPCS-Fe and NBrPCS-Fe. High-resolution XPS spectra of (**e**) B 1s for NBPCS-Fe and (**f**) Br 3d for NBrPCS-Fe.

Owing to the unique characteristics mentioned above, NSPCS possesses an innate ability to attract metal ions via electrostatic interactions, thereby serving as a desirable carbon support for the formation of well-dispersed Fe-N-C electrocatalyst. The Fe-N-C embedded NSPCS (NSPCS-Fe) can be synthesized by using NSPCS to adsorb ferroin, followed by pyrolysis in N_2_ atmosphere. The XRD pattern of NSPCS-Fe is similar to that of NSPCS, further demonstrating the absence of Fe-based aggregation (Figure 3a). SEM images of NSPCS-Fe display that the sphere-like morphology was maintained after adsorbing ferroin and subsequent carbonization. As shown in Figure 3c, no Fe aggregation can be observed in the carbon matrix. Notably, Figure 3d reveals that numerous worm-like mesopores are spread throughout the carbon matrix in NSPCS-Fe, which is beneficial for the full exposure of Fe-N-C active sites. The well-defined pore structure of NSPCS presents a confinement effect that prevents the aggregation of iron, resulting in a homogeneous distribution of Fe-N-C sites within NSPCS-Fe. EDS elemental mapping reveal that N, S, O, and Fe elements were uniformly distributed in the entire carbon framework (Figure 3e,f). Compared to NSPCS, the BET specific surface area and pore volume of NSPCS-Fe increase to 1325 m^2^ g^−1^ and 1.07 cm^3^ g^−1^, respectively (Figure 3b). In addition, the pore size distribution curve displays an increasing ratio of mesopores in NSPCS-Fe, consistent with the TEM results. Raman spectra reveal a higher *I*_D_/*I*_G_ ratio for NSPCS-Fe (0.87) compared to NSPCS(0.84), suggesting the increasing defects after the second carbonization (Figure S7a). XPS analysis indicates the existence of C, N, S, O, Si, and Fe in NSPCS-Fe, with mass contents calculated to be 80.6, 2.1, 2.1, 11.9, 2.0, and 1.3 wt%, respectively (Figure S10a and Table S1). The high-resolution N 1s spectra of NSPCS-Fe display the presence of five nitrogen species: pyridinic N at 398.4 eV, Fe-coordinated N (Fe-N) at 399.5 eV, pyrrolic N at 400.8 eV, graphitic N at 401.9 eV, and oxidized N at 403.5 eV (Figure S10b) [38]. It is recognized that the presence of pyridinic N and graphitic N species is advantageous for enhancing the diffusion-limited current density and expediting the four-electron transfer process during the ORR [39,40]. The high-resolution S 2p spectra in Figure S10c illustrate that the presence of thiophene-S in NSPCS-Fe originates from the carbon support, which is believed to increase the electron density on the Fe-N-C active sites, thereby enhancing the oxygen reduction reaction (ORR) activity [17,41]. The high-resolution Fe 2p spectra, as shown in Figure S10d, demonstrate that all Fe species present are in the form of Fe^2+^ (at 711.3 and 724.2 eV) and Fe^3+^ (at 715.4 and 726.1 eV). It is worth noting that no Fe^0^ peak was found, and the peak at 711.3 eV is attributed to Fe–N bonding, further demonstrating the existence of Fe-N-C active sites within NSPCS-Fe [42]. Different heteroatom-doped carbon support loading on Fe-N-C were carried out through our method. The SEM images of NPCS-Fe, NBPCS-Fe and NBrPCS-Fe, shown in Figure 4a–c, indicate that these samples exhibit similar sphere-like nanostructures. The average particle sizes of NSPCS-Fe, NPCS-Fe, NBPCS-Fe and NBrPCS-Fe are 350 nm, 260 nm, 110 nm and 180 nm, respectively (Figure S11). Interestingly, NSPCS-Fe exhibits a bigger particle size but higher ORR activity compared to NPCS-Fe, NBPCS-Fe and NBrPCS-Fe. As shown in Figure S12, no Fe-related diffraction peaks were detected in XRD patterns of NPCS-Fe, NBPCS-Fe and NBrPCS-Fe. The XPS survey spectra indicate the existence of C, N, O, Fe and Si in NPCS-Fe; C, N, O, Br, Fe and Si in NBrPCS-Fe; and C, N, O, B, Fe and Si in NBPCS-Fe, respectively (Figure 4d). The high-resolution N 1s spectra of these samples display five N species, which are same as for NSPCS-Fe (Figure S13a–c). The Fe–N bond in the high-resolution Fe 2p spectra of these samples further verifies the universality of our strategy (Figure S13d–f). Furthermore, the high-resolution B 1s spectrum of NBPCS-Fe can be divided into three peaks, B-N-C at 189.5 eV, BC_3_ at 191.6 eV and B-O at 192.5 eV, respectively (Figure 4e) [43]. The high-resolution Br 3d spectrum of NBrPCS-Fe is assigned to the C-Br bond at 69.0 eV (Figure 4f) [44].

**Figure 5:**
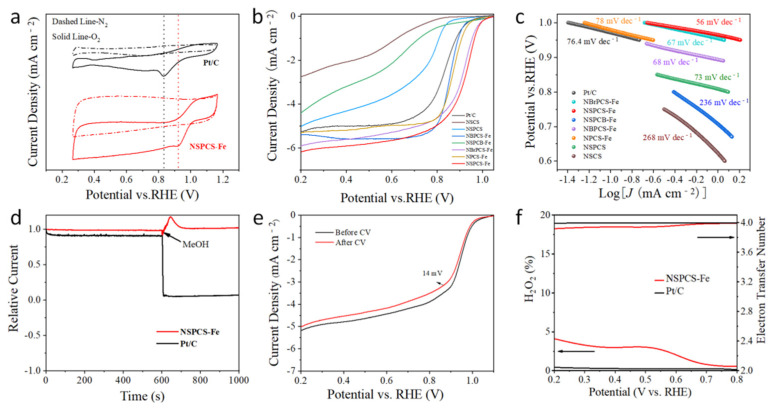
(**a**) CV curves of NSPCS-Fe and Pt/C. (**b**) ORR polarization curves of several NSPCS-Fe samples with different morphologies and chemical structures. (**c**) Corresponding Tafel plot. (**d**) Chronoamperometric responses of different catalysts after adding 10 mL methanol at 600 s. (**e**) ORR polarization curve of NSPCS-Fe before and after 3000 CV cycles. (**f**) H_2_O_2_ yield and electron transfer number from RRDE measurement.

The oxygen reduction reaction (ORR) activity of the NSPCS-Fe catalysts was tested using a rotating disk electrode in a 0.1 M potassium hydroxide (KOH) solution. Cyclic voltammograms (CV) were first studied in a 0.1 M KOH electrolyte saturated with oxygen gas or nitrogen gas (Figure 5a). The CV curves showed no significant redox peaks in the nitrogen-saturated electrolyte. In contrast, the NSPCS-Fe catalyst exhibited a more positive cathodic peak than the Pt/C catalyst when the electrolyte was oxygen-enriched. Linear sweep voltammetry (LSV) measurements were then performed on these electrocatalysts in an oxygen-saturated electrolyte, utilizing a rotation speed of 1600 rpm and a scan rate of 10 mV s^−1^. As shown in Figure 5b, the LSV curves indicate that the half-wave potential and limited current density of NSPCS-Fe can reach 0.91 V and 6.2 mA cm^−2^, which are much higher than those of Pt/C (0.83 V, 5.3 mA cm^−2^) and NSPCS (0.73 V, 5.0 mA cm^−2^), and comparable to most reported Fe-related electrocatalysts (Table S3). The Tafel plots derived from LSV curves reveal that NSPCS-Fe exhibits the lowest slope (56 mV dec^−1^) compared to NSPCS (73 mV dec^−1^) and the Pt/C catalyst (76 mV dec^−1^), indicating superior ORR activity for NSPCS-Fe (Figure 5c). This enhanced activity is attributed to its spherical architecture, unique pore structure and evenly distributed Fe-N-C active sites. To study the ORR performance of NSPCS-Fe more deeply, an LSV test was conducted with rotation speeds increasing from 400 to 2000 rpm in an oxygen-saturated electrolyte. The Koutecky-Levich (K-L) plots derived from LSV curves of NSPCS-Fe demonstrate good linearity and a similar slope to Pt/C at 0.55 V, suggesting first-order reaction kinetic behavior and a four-electron transfer mechanism for the ORR process (Figure S14) [45,46]. The ideal electrochemical reduction of oxygen involves a four-electron pathway leading to the formation of water. Nonetheless, a side reaction of two-electron reduction may occur, resulting in the formation of hydrogen peroxide (H_2_O_2_). To elucidate the ORR mechanism, a series of tests using a rotating ring disk electrode (RRDE) were conducted. Within the potential range of 0.2 to 0.9 V, the electron transfer number of the NSPCS-Fe catalyst was from 3.91 to 3.99, with the H_2_O_2_ yield consistently kept under 5%, comparable to that of the commercial Pt/C catalyst (Figure 5f). These results suggest that the NSPCS-Fe catalyst predominantly facilitates a direct four-electron reduction pathway for the oxygen reduction reaction. The ORR performance of NSPCB-Fe, NPCS-Fe, NBPCS-Fe and NBrPCS-Fe were tested to further study the effect of morphology and surface chemical structure. The LSV curves show that the half-wave potential and limited current density of NSPCB-Fe with bulk architecture are much lower than that of NSPCS-Fe, due to the low exposure area and sparse Fe-N-C active sites. The lower ORR kinetics were further demonstrated by the large Tafel slope of NSPCB-Fe (236 mV dec^−1^). Compared to NPCS-Fe, NXPCS-Fe (X = S, B, Br) possesses higher half-wave potential and limited current density, indicating that the surface chemical structure of the carbon support plays an important role in promoting the activity of Fe-N-C active sites. Among them, NSPCS-Fe reveals the highest half-wave potential and limited current density, due to the large atomic size and low electronegativity of S, which can modify the coordination environment of Fe-N-C sites. As shown in Figure 5c, the Tafel slope of NXPCS-Fe (S, Br or B) was much lower than that of NPCS-Fe, indicating that the introduction of a heteroatom (S, Br or B) into N-doped carbon support can promote ORR kinetics. These results demonstrate that, by optimizing the morphology and surface chemical structure of the carbon support, NSPCS-Fe, which features a spherical morphology, a unique pore structure, N,S-decoration, and uniformly dispersed Fe-N-C active sites, exhibits exceptional ORR activity.

It is important to highlight that durability and methanol tolerance are critical parameters in assessing the performance of ORR catalysts. As shown in Figure 5d and Figure S15, the current density of NSPCS-Fe exhibited only a 7% decrease after 10 h of ampero-metric i-t testing, and there was no significant change after methanol injection, which indicate the excellent durability and methanol tolerance of the NSPCS-Fe catalyst. In contrast, On the other hand, the commercial Pt/C catalyst displayed a rapid decline of 16% in current density after running for 10 h, and obvious fluctuations were observed after the addition of methanol. Additionally, the half-wave potential of NSPCS-Fe demonstrates a negligible negative shift of just 14 mV after 3000 CV cycles, further demonstrating its outstanding cycling stability (Figure 5e). Above all, the superior long-term stability and activity of NSPCS-Fe can be attributed to its unique structural characteristics, which include: (1) a spherical architecture with widespread worm-like mesopores that can efficiently expose the Fe-N-C active sites; (2) a large specific surface area and high microporosity that facilitate the rapid transport of ORR-related aqueous species and electrolyte ions; (3) the introduction of S into the N-doped carbon matrix, which can modify the electronic structure of the Fe-N-C sites and thus accelerate the entire ORR process.

## 3. Materials and Methods

### 3.1. Materials

Octaaminophenyl polyhedral oligomeric silsesquioxane (POSS-NH_2_) was purchased from Meryer (Shanghai, China). Isophthalaldehyde, 3,5-diformylphenylboronic acid, 2,5-thiophenedicarboxaldehyde, 2-bromobenzene-1,3-dialdehyde, ethanol, methanol, tetrahydrofuran (THF), 1,2-dichloroethane, hydrofluoric acid (HF), ferrous acetate, 1,10-Phenanthroline and potassium hydroxide were purchased from Macklin (Shanghai, China). Acetone and acetic acid were acquired from Guangzhou Chemical Reagent Company (Guangzhou, China). A 0.5 wt% Nafion solution was acquired from Sigma-Aldrich (St. Louis, MO, USA). 20%Pt/C was obtained from Alfa Aesar (Haverhill, MA, USA). All chemicals were directly used without further purification.

### 3.2. Synthesis

#### 3.2.1. Synthesis of NSPCS

In a typical synthesis procedure, 0.4 mmol of 2,5-thiophenedicarboxaldehyde was dissolved in 1mL of methanol to form a clear solution, which was then added to 1 mL of methanol containing 0.1 mmol of POSS-NH_2_ and 0.1 mL of acetic acid. N,S-codoped polymer sphere (NSPS) could be obtained after polymerization for 10 min, followed by filtration and vacuum drying at 60 °C. Then, the as-prepared NSPS was heated to 900 °C (at 5 °C min^−1^) for 5 h in a steam of N_2_ to form an N,S-codoped carbon sphere (NSCS). Finally, the target N,S-codoped porous carbon sphere (NSPCS) was acquired by etching NSCS with HF (20%) for 12 h. Syntheses of N doped porous carbon sphere (NPCS), N,B-codoped porous carbon sphere (NBPCS) and N,Br-codoped porous carbon sphere (NBrPCS) were the same as for NSPCS, but using isophthalaldehyde, 3,5-diformylphenylboronic acid and 2-bromobenzene-1,3-dialdehyde, respectively. Syntheses of N,S-codoped porous carbon bulk (NSPCB) were the same as for NSPCS but used THF as the polymerization solvent.

#### 3.2.2. Synthesis of NSPCS-Fe

Typically, 30 mg of 1,10-Phenanthroline and 10 mg of ferrous acetate (Fe(Ac)_2_) were dispersed in 40 mL of water and sonicated for 30 min to form ferroin (Fe(Phen)_3_^2+^) solution. Then, 50 mg of NSPCS were added into the solution and stirred for 12 h at room temperature. Afterwards, the products were collected and vacuum dried at 60 °C. NSPCS-Fe can be obtained after annealing the products at 1000 °C for 1 h. For comparison, NPCS-Fe, NBPCS-Fe, NBrPCS-Fe and NSPCB-Fe were prepared under identical conditions.

### 3.3. Characterization

The morphology of samples were characterized by an S-4800 field emission scanning electron microscope (FESEM, Hitachi, Tokyo, Japan) and an FEITecnai G2 f20s-twin field emission transmission electron microscope(FETEM, FEI, Hillsboro, OR, USA). X-ray photoelectron spectroscopy (XPS) was performed on a Thermo ESCALAB 250 X-ray photoelectron spectrometer. X-ray diffraction (XRD) was detected on a D8 ADVANCE. Fourier-transform infrared (FT–IR) was conducted via Bruker TENSOR 27 infrared spectroscopy (Bremen, Germany) by a KBr disk method. Thermogravimetric analysis (TGA, Woden, Australia, TG-209/Vector-22) was carried out from 100 °C to 800 °C with a heating rate of 10 °C min^−1^ under oxygen or nitrogen atmosphere. The Raman spectra were determined by a HORIBA (Kyoto, Japan) JY LabRAM HR Evolution with 514 nm laser. N_2_ adsorption–desorption isotherm and the corresponding pore-size distribution was recorded on a Micromeritics ASAP2020 analyzer.

### 3.4. Electrochemical Test

The oxygen reduction reaction (ORR) activities of all samples were evaluated in a 0.1 M KOH electrolyte, utilizing a conventional three-electrode setup on a CS2350M workstation (Corrtest Instruments, Wuhan, China). In the conventional three-electrode system, a platinum plate served as the counter electrode, an Ag/AgCl electrode functioned as the reference, and the GCE loaded with the catalyst acted as the working electrode. For the catalyst ink formulation, 5 mg of the active materials was ultrasonically dispersed in a mixed solvent comprising 500 μL of ethanol, 450 μL of deionized water, and 50 μL of a 5 wt% Nafion solution, for a duration of 30 min to ensure a homogeneous dispersion. This process yielded a stable ink, which was then applied to a glassy carbon electrode (GCE) and allowed to dry under ambient conditions. The mass loading of the catalyst on the electrode was standardized at 0.48 mg cm^−2^, whereas a commercial Pt/C catalyst was applied at a loading of 0.2 mg cm^−2^ for comparative analysis.

In the ORR testing, cyclic voltammetry (CV) measurements were conducted between 0.26 V and 1.16 V at a scan rate of 20 mV s^−1^ in an electrolyte saturated with N_2_ or O_2_. Linear sweep voltammetry (LSV) data were obtained in an O_2_-saturated electrolyte at a scan rate of 10 mV s^−1^, with the rotating disk electrode (RDE) and rotating ring disk electrode (RRDE) speeds ranging from 400 to 2000 rpm. For the stability test, the LSV test was carried out before and after 3000 CV cycles, and the CV was measured at a scan rate of 20 mV s^−1^ over a potential range from −0.3 to 0.1 V. The durability and methanol crossover tolerance of the catalysts was evaluated using ampero-metric i-t measurements at a constant rotation speed of 600 rpm in O_2_-saturated electrolyte. All reported electrode potentials were referenced against a reversible hydrogen electrode (RHE) to maintain consistency with standard electrochemical practices. The measured potentials versus Ag/AgCl were converted to a reversible hydrogen electrode (RHE) scale according to the Nernst equation (E_RHE_ = E_Ag/AgCl_ + 0.059×pH + 0.197).

## 4. Conclusions

In summary, a novel synthetic method for the fabrication of a carbon support with tunable morphology and customizable surface chemical structure was developed to achieve efficient ORR activity of a Fe-N-C electrocatalyst. The large specific surface area and well-defined pore structure of as-prepared NSPCS originate from the removal of homogeneously dispersed nano-silica domains. The morphology and surface chemical structure can be precisely tailored by selecting proper solvent and heteroatom-containing aldehydes. Benefiting from the sphere-like morphology, well-defined pore structure, large specific surface area and efficient N,S-codoping, NSPCS-Fe exhibits excellent ORR activity and durability, which is superior to the commercial Pt/C catalyst and comparable to other reported Fe-based electrocatalysts. This study not only advances the field of electrocatalysis but also offers an alternative method for the fabrication of well-designed carbon materials for many applications in the energy field.

## Data Availability

The original contributions presented in the study are included in the article (and Supplementary Materials), further inquiries can be directed to the corresponding authors.

## Supplementary Materials

The following supporting information can be downloaded at: https://www.mdpi.com/article/10.3390/molecules29163785/s1, Figure S1: (a) FT-IR spectra of NSPS. (b) Raman spectrum of NSPS; Figure S2: (a) SEM image of NSPS. (b) Size distribution of NSPS; Figure S3: XRD pattern of NSPS; Figure S4: (a) SEM images of NSCS. (b) TGA curves of NSPS; Figure S5: TGA curves of NSCS and NSPCS under O_2_ atmosphere; Figure S6: XRD patterns of NSCS and NSPCS; Figure S7: Raman spectra of (a) NSCS, NSPCS and Fe-NSPCS; (b) NPCS, NSPCB, NBPCS and NBrPCS.; Figure S8: SEM images of NSPCS using (a) THF, (b) ethanol, and (c) acetone as the polycondensation solvents; Figure S9: (a) Nitrogen adsorption-desorption curves of NPCS, NBPCS and NBrPCS and (b) corresponding pore size distribution; Figure S10: (a) XPS summary spectrum, (b) high-resolution N 1s, (c) high-resolution S 2p and (d) high-resolution Fe 2p of NSPCS-Fe; Figure S11. The particle size distribution of (a) NSPCS-Fe, (b) NPCS-Fe, (c) NBPCS-Fe and (d) NBrPCS-Fe; Figure S12: XRD patterns of NPCS-Fe, NBPCS-Fe, NBrPCS-Fe and NSPCB-Fe; Figure S13: High-resolution (a) N 1s and (d) Fe 2p of NPCS-Fe. High-resolution (b) N 1s and (e) Fe 2p of NBPCS-Fe. High-resolution (c) N 1s and (f) Fe 2p of NBrPCS-Fe; Figure S14: (a) K-L plots of the samples at different rotating speed and a potential of 0.55 V. K-L plots of (b) Pt/C, (c) NSPCS-Fe, (d) NPCS-Fe, (e) NSPCS, (f) NSPCB-Fe, (g) NBPCS-Fe and (h) NBrPCS-Fe derived from LSV curves at different rotating speed and various potential; Figure S15: Chronoamperometric curves of NSPCS-Fe and Pt/C at a potential of 0.57 V in an O_2_-saturated 0.1 M KOH solution. Table S1: Element contents of the samples; Table S2: BET specific surface area and pore volumes of the samples; Table S3: Comparison of ORR catalytic activity between NSPCS-Fe and other Fe-based electrocatalysts in 0.1 M KOH solution.
