# Supplementary material for: Precisely Designed Morphology and Surface Chemical Structure of Fe-N-C Electrocatalysts for Enhanced Oxygen Reaction Reduction Activity
Source: Molecules. 2024 Aug 10;29(16):3785. doi: 10.3390/molecules29163785 (PMC11357191; doi:10.3390/molecules29163785)
Supplement: Supplementary file 1 [file molecules-29-03785-s001.zip › molecules-3118821-supplementary.pdf]

Supporting Information

Precisely designed morphology and surface chemical structure of Fe-N-C electrocatalysts for Enhanced oxygen reaction reduction activity

Zirun Chen*, Yuhao Xiong, Yanling Liu, Zhanghongyuan Wang, Binbin Zhang, Xingtang Liang, Xia Chen and Yanzhen Yin*

Guangxi Key Laboratory of Green Chemical Materials and Safety Technology，Beibu Gulf University, Qinzhou 535011, China.

Email: chenzr@bbgu.edu.cn (Z. R.); yinyanzhen@bbgu.edu.cn (Y. Y.); Tel.:+86-182-9019-3615 (Z. R.) ; +86-182-9019-3615 (Z. R.)

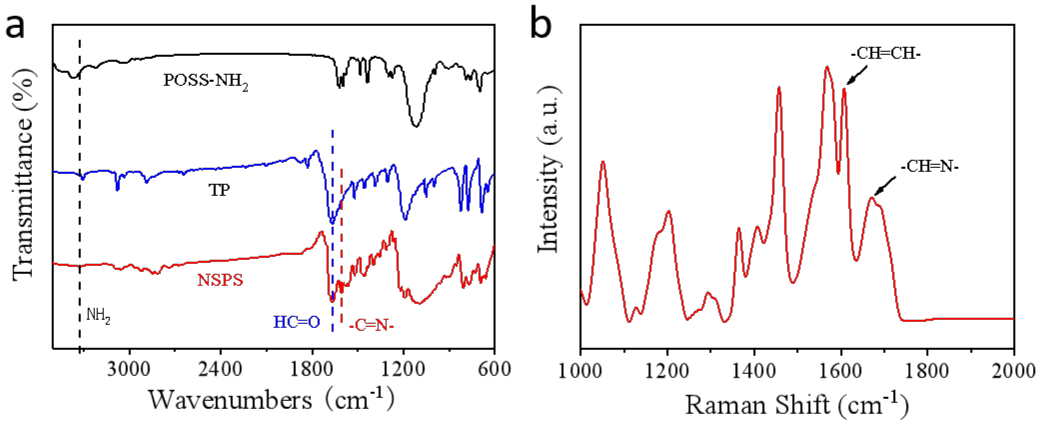

Figure S1. (a)FT-IR spectra of NSPS. (b) Raman spectrum of NSPS.

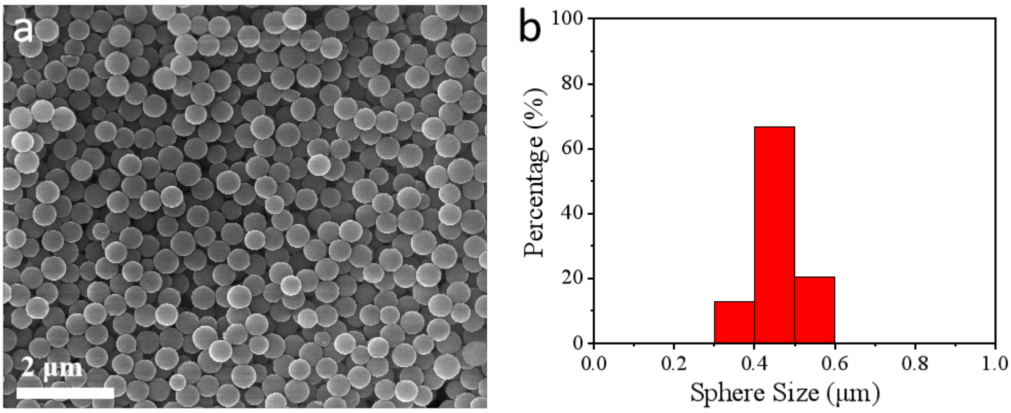

Figure S2. (a) SEM image of NSPS. (b) Size distribution of NSPS.

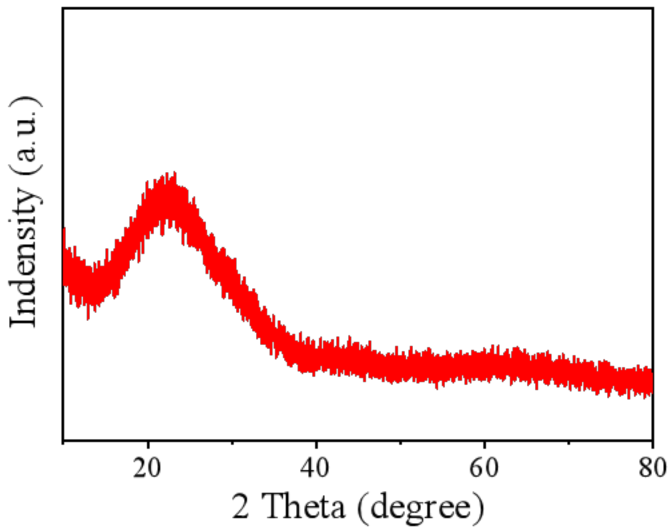

Figure S3. XRD pattern of NSPS.

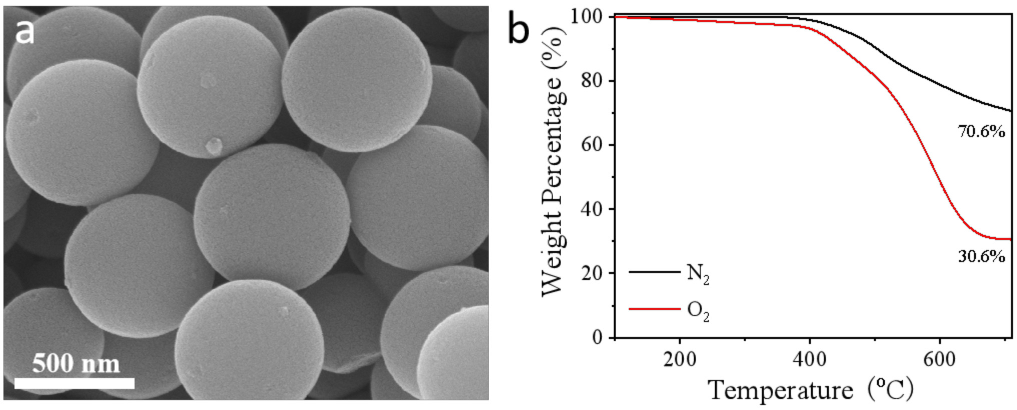

Figure S4. (a) SEM image of NSCS. (b) TGA curves of NSPS.

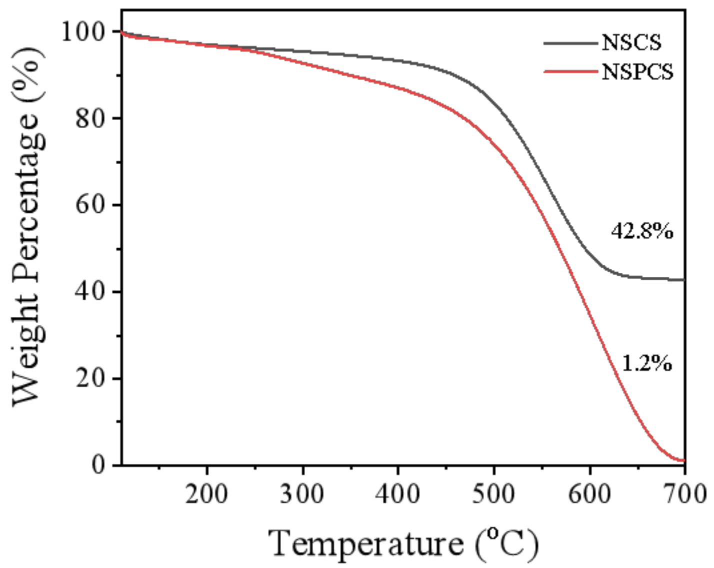

Figure S5. TGA curves of NSCS adn NSPCS under O_2 atmosphere.

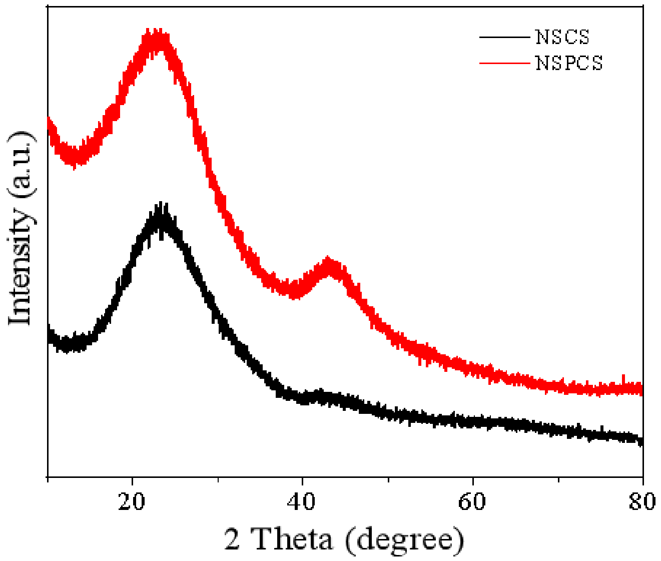

Figure S6. XRD patterns of NSCS and NSPCS.

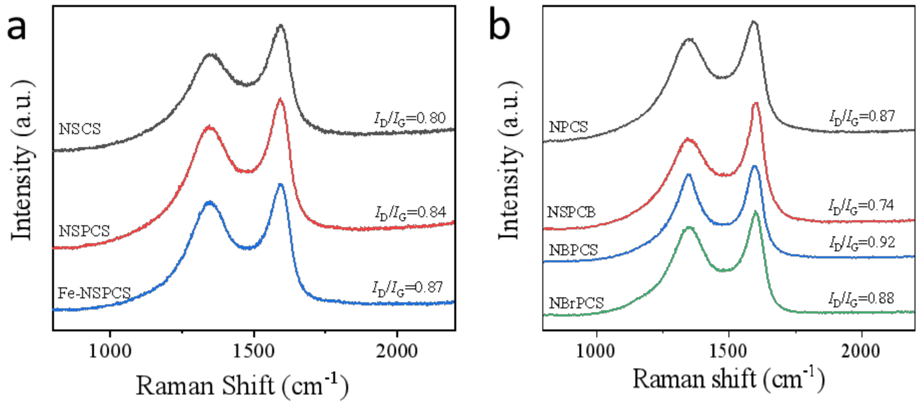

Figure S7. Raman spectra of (a) NSCS, NSPCS and Fe-NSPCS; (b) NPCS, NSPCB, NBPCS and NBrPCS.

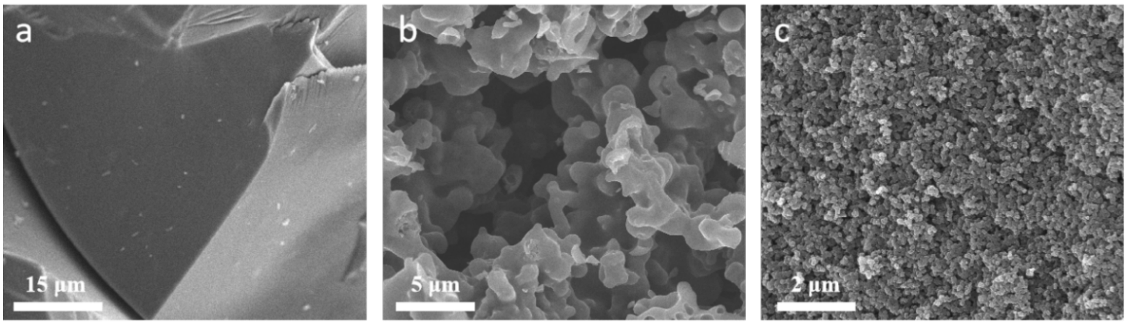

Figure S8. SEM images of NSPCS using (a) THF, (b) ethanol, and (c) acetone as the polycondensation solvents.

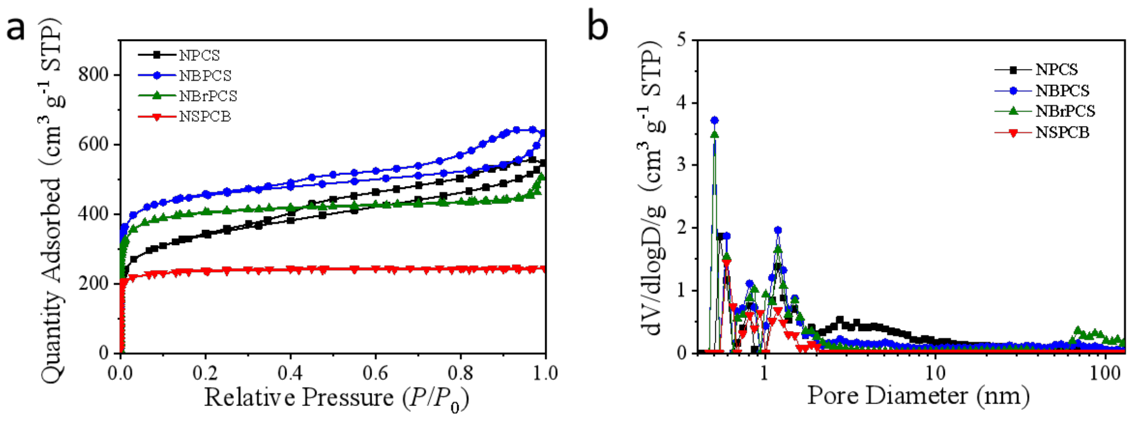

Figure S9. (a) Nitrogen adsorption-desorption curves of NPCS, NBPCS and NBrPCS and (b) corresponding pore size distribution.

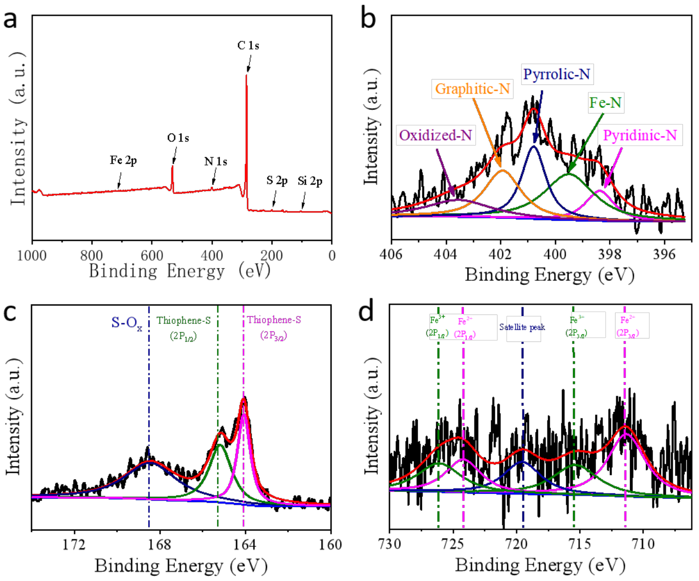

Figure S10. (a) XPS summary spectrum, (b) high-resolution N 1s, (c) high-resolution S 2p and (d) high-resolution Fe 2p of NSPCS-Fe.

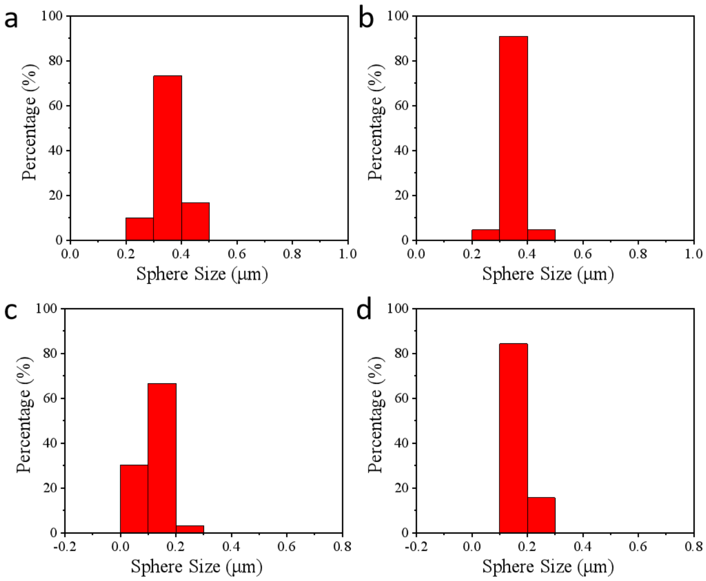

Figure S11. The particle size distribution of (a) NSPCS-Fe, (b) NPCS-Fe, (c) NBPCS-Fe and (d) NBrPCS-Fe.

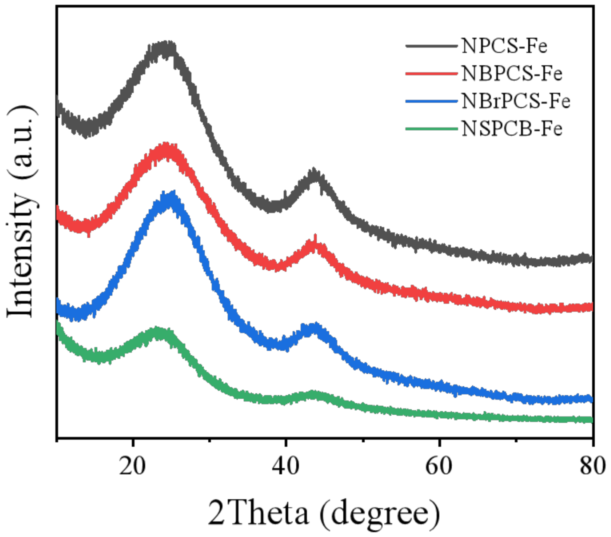

Figure S12. XRD patterns of NPCS-Fe, NBPCS-Fe, NBrPCS-Fe and NSPCB-Fe.

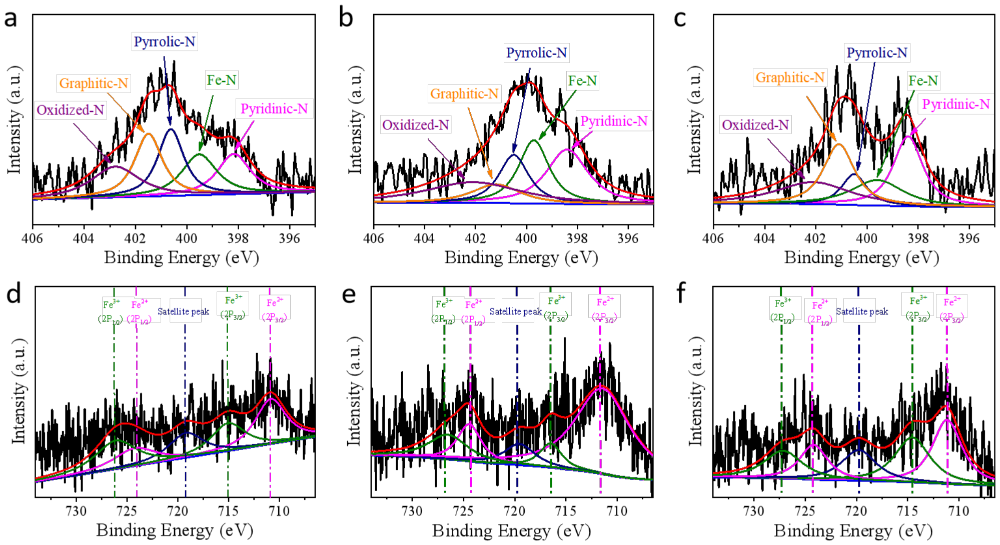

Figure S13. High-resolution (a) N 1s and (d)Fe 2p of NPCS-Fe. High-resolution (b) N 1s and (e)Fe 2p of NBPCS-Fe.High-resolution (c) N 1s and (f)Fe 2p of NBrPCS-Fe.

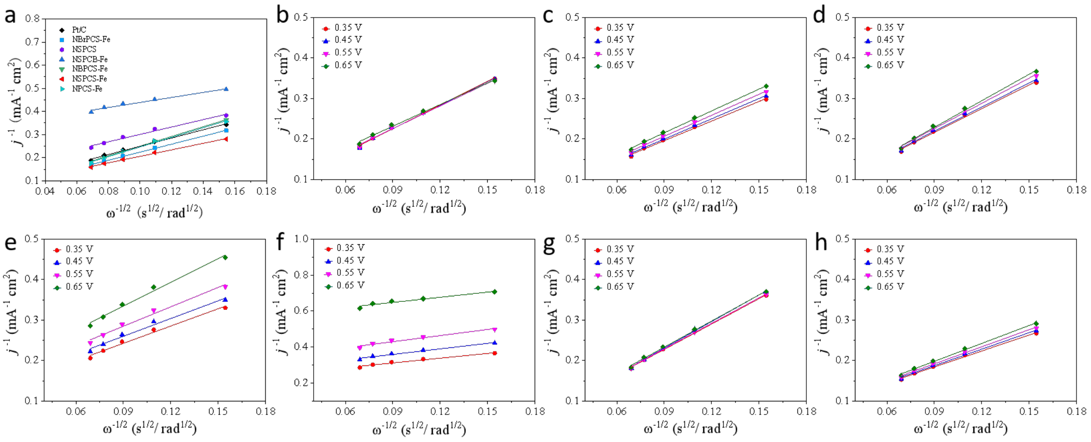

Figure S14. (a) K-L plots of the samples at different rotating speed and a potential of 0.55 V. K-L plots of (b) Pt/C, (c) NBrPCS-Fe, (d) NPCS-Fe, (e) NSPCS, (f) NSPCB-Fe, (g) NBPCS-Fe and (h) NSPCS-Fe derived from LSV curves at different rotating speed and various potential.

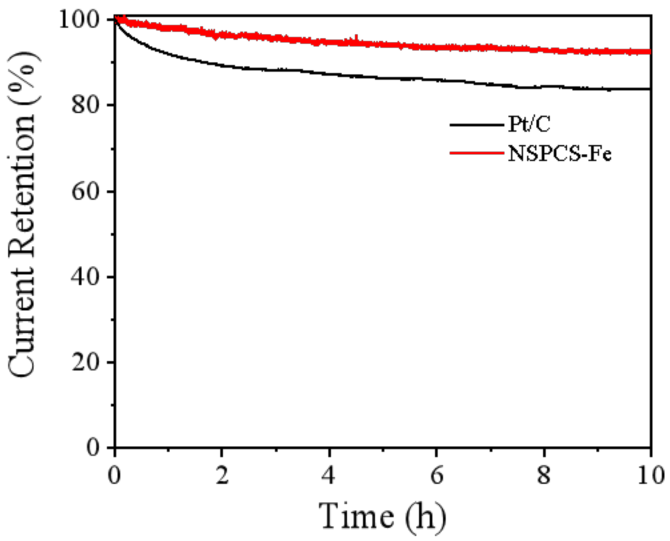

Figure S15. Chronoamperometric curves of NSPCS-Fe and Pt/C at a potential of 0.57 V in an O_2-saturated 0.1 M KOH solution.

Table S1. Element contents of the samples.

Samples	C content (at%)	N content (at%)	O content (at%)	Si content (at%)	S content (at%)	Fe content (at%)	Br content (at%)	B content (at%)
NSCS	55.7	2.1	29.6	11.4	1.2	/	/	/
NSPCS	80.5	3.0	15.3	0.3	0.9	/	/	/
NBPCS	88.4	2.5	5.8	1.1	/	/	/	2.2
NBrPCS	90.9	1.9	5.3	1.9	/	/	0.1	/
NSPCS-Fe	84.5	2.7	9.7	1.1	1.8	0.3	/	/
NPCS-Fe	90.9	1.1	7.0	0.8	/	0.2	/	/
NBPCS-Fe	84.3	2.3	9.0	0.8	/	0.3	/	3.4
NBrPCS-Fe	90.3	1.3	6.9	0.9	/	0.4	0.1	/

Table S2. BET specific surface area and pore volumes of the samples.

Samples	S_{BET} ($m^2\ g^{-1}$)	$S_{micropore}$ ($m^2\ g^{-1}$)	$S_{mesopore}$ ($m^2\ g^{-1}$)	Pore volume ($cm^3\ g^{-1}$)
NSCS	166	110	56	0.17
NSPCS	1114	787	326	0.61
NSPCS-Fe	1325	518	807	1.07
NPCS	1173	516	657	0.85
NBPCS	1531	1091	440	0.98
NBrPCS	1362	1050	312	0.78
NSPCB	784	670	114	0.38

Table S3. Comparison of ORR catalytic activity between NSPCS-Fe and other Fe-based electrocatalysts in 0.1 M KOH solution.

Catalyst	$E_{1/2}$ (V)	Reference
NSPCS-Fe	0.91	This work
NiFe-N-C	0.87	Energy Environ. Sci. **2024**, 17, 704-716.
NC@Fe_3C-900	0.88	*Adv. Funct. Mater.* **2024**, 2403810.
CoSAC@FePc	0.90	*Adv. Funct. Mater.* **2024**, 2314554.
Fe–N_4SP/NPS-HC	0.91	*Energy Environ. Sci.* **2024**, 17, 249-259.
FePNC	0.90	*Adv. Energy Mater.* **2023**, 13, 2301223.
FePc-{PW12}@NT	0.90	*Angew. Chem. Int. Ed.* **2023**, 62, e202309545.
Fe-ACSA@NC	0.90	*Angew. Chem. Int. Ed.* **2022**, 61, e202116068.
Fe/Meso-NC-1000	0.89	*Adv. Mater.* **2022**, 34, 2107291.
SA&NP-FeCo-NTS	0.87	*Adv. Funct. Mater.* **2022,** 32, 2112805.
FeCo-DACs/NC	0.88	*Adv. Mater.* **2022**, *34*, 2107421.
Fe/Meso-NC-1000	0.89	*Adv. Mater.* **2022**, *34*, 2107291.
